# A Comparison of Face to Face and Video-Based Self Care Education on Quality of Life of Hemodialysis Patients

**Published:** 2015-07

**Authors:** Masumeh Hemmati Maslakpak, Shadi Shams

**Affiliations:** 1 Department of Medical Surgical Nursing, Maternal and Childhood Obesity Research Center, Urmia University of Medical Sciences, Urmia, Iran;; 2 Department of Medical Surgical Nursing, Urmia University of Medical Sciences, Urmia, Iran

**Keywords:** Hemodialysis, Patient education, Quality of life, Self-care

## Abstract

**Background:**

End stage renal disease negatively affects the patients’ quality of life. There are different educational methods to help these patients. This study was performed to compare the effectiveness of self-care education in two methods, face to face and video educational, on the quality of life in patients under treatment by hemodialysis in education-medical centers in Urmia.

**Methods:**

In this *quasi-experimental *study, 120 hemodialysis patients were selected randomly; they were then randomly allocated to three groups: the control, face to face education and video education. For face to face group, education was given individually in two sessions of 35 to 45 minutes. For video educational group, CD was shown. Kidney Disease Quality Of Life- Short Form (KDQOL-SF) questionnaire was filled out before and two months after the intervention. Data analysis was performed in SPSS software by using one-way ANOVA.

**Results:**

ANOVA test showed a statistically significant difference in the quality of life scores among the three groups after the intervention (P=0.024). After the intervention, Tukey’s post-hoc test showed a statistically significant difference between the two groups of video and face to face education regarding the quality of life (P>0.05).

**Conclusion:**

Implementation of the face to face and video education methods improves the quality of life in hemodialysis patients. So, it is suggested that video educational should be used along with face to face education.

## Introduction

End Stage Renal Disease (ESRD) is one of the major health problems worldwide.^1^ Patients with end stage renal disease are growing because of rising diseases such as diabetes, hypertension and malignancies.^2^ The prevalence rate of end-stage renal disease (ESRD) has increased by 8% from 2007 to 2012.^3^ In 2008, over 16600 patients with ESRD were under treatment with maintenance hemodialysis (HD) in 355 dialysis units in Iran.^4^

End stage renal disease and therapeutic approaches about it, such as hemodialysis, influence lifestyle, health status and the roles of the person. Even though hemodialysis causes improvement of health and enhances the patient’s survival, disease process has not changed and it is not entirely substituted by renal function.^5^^,^^6^ In long-term, it will lead to a decline in living standards, physical and psychological problems and limitations in recreational, social and occupational activities.^7^ Numerous studies conducted in various countries indicate that patients undergoing HD have lower quality of life in comparison to the healthy population.^8^^,^^9^ Lower-than-normal quality of life in hemodialysis patients is an important problem in these patients.^10^ Several factors are involved in reducing the quality of life in these patients; they include stress,^11^ depression and anxiety,^12^ anemia,^13^ hospitalization,^14^ and inactivity.^13^ Quality of life in people with chronic disease is related to their individual characteristics and it depends on people’s coping skills in different situations of life.^15^

Promotion of the life quality is considered as one of the major goals in treatment of chronic patients. Considering the chronic and debilitating end stage of renal diseases and the need to long-term use of hemodialysis, the need to education for improving the quality of life in these patients seems to be required.^16^ Patient education is one of the essential aspects of nursing activity which might cause health improvement, complication prevention and patient quality of life promotion.^17^^,^^18^ Education on self-care behaviors in patients treated with hemodialysis including control of fluid intake, food and medicinal regime, involvement in the care, effective communication leads to self-efficacy and role preservation and also causes improvement in quality of life in these patients.^19^

There are different educational methods to help patients to comply with lifestyle changes.^20^ Face to face education is one of the most common methods of training in the health care system. Such education helped patients manage their conditions; it has also provided the opportunity for the patient to ask questions, and the patient could discuss any question concerning the issue to the nurse, and modified inaccurate beliefs and information in their mind. So, it reduced the patient’s concerns; on the other hand, the nurse was assured about his understanding of information by face to face contact with the patient and a dynamic relationship is made between the patient and the nurse.^21^ But, much more time is needed for education and it is not possible in overcrowded centers. One of the other disadvantages of this method in hemodialysis patients is the problems about implementing these educational programs during dialysis and inviting patients for education at other times is difficult.^22^

Therefore, finding effective educational methods that can solve the problems and can be used between the dialysis sessions is essential and requires research.^23^ Advancement of communication technology and expanding various communication methods and tools have provided the possibility of using video education. The advantages of video education are the ability to create storage and continuity of information and it is easy to use and cost-effective. But besides these advantages, one of its most important weaknesses is being virtual and lack of a lively, active educator in the program implementation; setting up active communication and reality plays an undeniable part in reaching the goal of teaching. Today, with the tremendous advances made in preparing educational films, these imperfections are also decreasing.^24^ Many studies have been conducted throughout the world addressing the issue of education, which have aimed at improving the health related quality of life in HD patients; these studies have mostly used oral or monitoring modalities.^25^^,^^26^ On the other hand, results of Baraz et al.’s study (2014) showed that oral and video education could affect the health related quality of life dimensions in the patients and improve their quality of life.^27^ So, this study aimed to compare the effectiveness of two methods of face to face and video self-care education on the quality of life in hemodialysis patients in educational hospitals of Urmia in 2013.

## Materials and Methods

This quasi-experimental, pretest-posttest interventional study was conducted in the dialysis units in two major general hospitals including Imam Khomeini and Ayatollah Taleghani affiliated with the Urmia University of Medical Sciences. These hospitals were governmental referral centers. The data were collected from August to December 2013 in Imam Khomeini with 105 patients and Ayatollah Taleghani with 135 patients under HD. Inclusion criteria for this study included age of 18-65 years, having at least the ability to read and write, not having hearing and vision impairments, being at least 6 months on hemodialysis, and not participating in any educational class out of the routine ones for at least during the last year. Exclusion criterion was not undergoing kidney transplant surgery during the study. The sample size, by considering type I error of 0.05 and type II error of 0.2 and based on mean difference of 5.5 and standard deviation of 0.55 in Hasanzade et al.’s study,^28^ was determined 80 patients for the intervention groups (video and face to face education). For the the control group (the equivalent of the number of one intervention group), 120 samples were considered for this study. The sampling method was simple random sampling which was proportionate to the population size, in two teaching hospitals of Imam Khomeini and Ayatollah Taleghani. From 240 hemodialysis patients in the two teaching medical centers, 167 patients had inclusion criteria for this study and a number was given to each patient. Each number was placed in a bowl and mixed thoroughly; the blind-folded researcher then picked the numbered tags from the bowl.

After obtaining theapproval by the ethics committee of Urmia University of Medical Sciences and meeting with the patients, explaining about this research and obtaining written consent from the patients, they were assured that all information provided by them will remain confidential and the study results will be presented anonymously. They were also informed that at every stage of research, they can stop their cooperation and leave the studyin case they are willing. After a needs assessment with the patients under hemodialysis treatment by interview, educational and caring needs of these patients were identified and educational content based on the needs assessments were decided: obeying dietary regimen, daily weight control, how to use medications properly, and coping with new life conditions. Kidney Disease Quality Of Life- Short Form questionnaire^29^^,^^30^ was given to the participants to complete before the intervention.

KDQOL-SF contains SF-36 questionnaire items along with other purposeful and specific items to evaluate aspects of life in renal patients. The latest version of it that was used in this study included 43 items on quality of life for renal patients and 36 items on general health. Specific dimensions of the questionnaire included symptoms and problem lists (12 items), effect of renal disease (8 items), burden of renal disease (4 items), occupational performance (2 items), cognitive performance (3 items), quality of social relationships (3 items), sexual performance (2 items,) sleep (4 items), and social support (2 items), medical staff support (2 items) and general health status (1 item). General dimensions included physical performance (10 items), restrictions about physical problems (4 items), physical pain (2 items), an understanding of general health (5 items), psychological health (5 items), limitations caused by emotional problems (3 items), social performance (2 items), liveliness (4 items) and patient satisfaction (1 item). Each dimension’s score ranged from zero to one hundred, with the higher scores showing better quality of life.^31^ In Fardinmehr et al.’s study, construct validity of the questionnaire was assessed by evaluating the correlation between each dimension’s score and the overall score of health using Pearson’s correlation coefficient. In that study, the reliability of the tool was found by Cronbach’s alpha to be 0.85.^29^ Yekaninezhad et al.’s study showed good test–retest reliability (all above 0.7). All of the items met the minimal criteria (i.e. 0.7 ) for internal consistency and Cronbach’s α ranged from 0.73 to 0.93. To test the construct validity, they examined correlations of the kidney disease targeted scales and overall health rating scale. The majority of items correlated significantly with the overall health rating.^30^ The alpha coefficient of eight subscales obtained in the present study was >0.75.

All questionnaires were filled out through individual interviews in a private room in the hemodialysis centers. A trained interviewer who was blind to the patients’ groups conducted all the interviews. The questions were asked by the interviewer in a simple and clear way and their answers were entered into the study instrument. After completing the questionnaires, 120 patients were randomly assigned into three groups including face-to-face education, video education and control groups by block randomization. The content of the educational material in two methods of face to face and video education was similar and was prepared considering patients educational needs through consultation with nephrologists and dieticians. The program aimed to enable hemodialysis patients to care for themselves in the domains of diet, fluid intake, fistula care, skin care, and stress management. Therefore, two educational programs were designed. After selecting the three groups, 40 patients received face to face education using educational booklet and 40 patients were given prepared CDs with the same educational content of the booklets. 40 patients were also in the control group who received routine care.

In the face to face educational group, an individual session education (2 education) was done over 35 to 45 minutes. One session per week was run by researchers. A classroom in the hemodialysis centers was considered for face to face educationa on the days after their hemodialysis sessions. Finally, totally 80 educational sessions were held for 40 patients in the face to face educational group. All training sessions were facilitated by both authors. A teaching booklet was prepared by the researchers and given to each patient at the end of the face to face educationa. The content validity of the booklet was confirmed by 9 faculty members in nursing school of Urmia. 

Patients in the video education group watched an educational film on a video disc during two consecutive dialysis sessions in a week. First, the patient was allowed to go to hemodialysis and after ensuring that the patient is stable and ready (usually following one to two hours after initiation of hemodialysis), he/she was invited to watch the 45-minute film. It was prepared by the researchers. The content validity of the CD was confirmed by 9 faculty members in nursing school of Urmia.

Two months after the education, KDQOL-SF questionnaire was completed again by the patients. Collected data were analyzed in SPSS software, version 20, using descriptive statistics and ANOVA. 

## Results

ANOVA and Chi-square test results showed that qualitative variables of gender, marital status, occupation, education, income, the number of dialysis a week and quantitative variables of age and duration of disease among the three groups were not significantly different. Mean and standard deviation of age and disease duration of the samples were 47.03±12.88, 4.14±4.89, respectively (Table 1).

**Table 1:** Comparison of demographic characteristics between three groups of control, face to face and video education

**Variable**	**Category**	**Control group**	**Video education group**	**Face to face education group**	**Chi-square test result**
		**N (%)**	**N (%)**	**N (%)**	
Gender	Woman	13 (36.1)	12 (33.3)	11 (30.6)	x^ 2 ^=0.238 df.=2 P=0.888
	Man	27 (32.1)	28 (33.3)	29 (34.5)	
Marital Status	Single	9 (42.9)	8 (38.1)	4 (19.0)	x^ 2 ^=2.424 df.=2 P=0.298
	Married	31 (31.3)	32 (32.3)	36 (36.4)	
Occupation	Retired	4 (18.2)	6 (27.3)	12 (54.5)	x^ 2 ^=17.034 df.=10 P=0.074
	Self-employment	18 (27.6)	13 (44.8)	8 (27.6)	
	Housewife	8 (27.4)	10 (36.3)	10 (36.3)	
	Unemployed	3 (25.0)	7 (58.3)	2 (16.7)	
	Employee	4 (33.3)	4 (33.3)	4 (33.3)	
	Disabled	3 (42.9)	0 (0.0)	4 (57.1)	
Education Level	Primary	15 (34.1)	16 (36.4)	13 (29.5)	x^ 2 ^=7.318 df.=6 P=0.292
	Secondary school	3 (16.7)	6 (33.3)	9 (50.0)	
	High school	16 (41.0)	9 (23.1)	14 (35.9)	
	Collegiate	6 (31.6)	9 (47.4)	4 (21.1)	
Income (Rial)	None	19 (44.2)	14 (32.6)	10 (23.3)	x^ 2 ^=11.227 df.=10 P=0.336
	Less than 3000,000.0	3 (37.5)	2 (25.0)	3 (37.5)	
	300 to 600,000,0	2 (11.8)	6 (35.3)	9 (52.9)	
	600 to 800,000,0	5 (23.8)	10 (47.6)	6 (28.6)	
	800,000,0 to 10,000,000	8 (34.8)	7 (30.4)	8 (34.8)	
	10,000,000 and above	3 (37.5)	1 (12.5)	4 (50.0)	
The Number of Dialysis per Week	Twice	4 (33.3)	5 (41.7)	4 (25.0)	x^ 2 ^=0.556 df.=2 P=0.757
	Three times	36 (33.3)	35 (32.4)	37 (34.3)	
Variable		Mean and standard deviation	Mean and standard deviation	Mean and standard deviation	ANOVA test result
Age (year)		45.75±14.04	46.33±12.29	49.03±12.32	P=0.483
Disease Duration (year)		3.77±3.87	4.50±5.61	4.15±5.13	P=0.806

Before the intervention, the mean quality of life scores in the three groups of control, video, and face to face had no statistically significant differences (P=0.376). Mean scores of quality of life after teaching the self-care in the video and face to face raised compared to the control group; this rise was significantly different (P=0.024). One-way ANOVA test for the independent three groups showed statistically significant differences between the mean scores of quality of life among the three groups after the intervention (P<0.001). The result of the ANOVA test showed that before the intervention the mean score for general health among three groups was not statistically different (P=0.429). But, after the intervention, the mean in all three groups had significantly different (P=0.001). The result of the ANOVA test showed that the mean scores of changes before and after the intervention for general health were different in the three groups (P=0.034) (Table 2).

**Table 2:** Mean comparison of quality of life and general health scores among three groups of control, face to face and video education, before and after the self-care program implementation

		**Control group**	**Video education group**	**Face to face education group**	**ANOVA test result** **P value**
		**Mean±SD**	**Mean±SD**	**Mean±SD**	
Quality of life	Before the intervention	56.94±19.887	55.45±18.733	51.73±11.309	0.376
	After the intervention	57.39±15.390	65.82±16.035	63.10±9.223	0.024
	Changes beforer - after	-0.45±15.567	-10.37±12.04943	-11.37±10.706	<0.001
General health	Before the intervention	41.50±19.82	47.21±23.05	45.21±16.04	0.429
	After the intervention	44.57±17.46	59.91±19.59	52.96±16.23	0.001
	Changes beforer - after	-3.07±13.04	-12.7±20.14	-7.75±15.02	0.034

Tukey’s post-hoc test showed that after the intervention, the changes in the mean score before and after the intervention in the quality of life for patients in the control group compared with the two groups of video and face to face education were a statistically significant (P<0.05). But, the changes in the mean score after and before it in the quality of life for patients between two, video and face to face education groups, did not show a statistically significant difference (P>0.05). Tukey’s post-hoc test showed that the mean scores of changes before and after it in general health between two, control and video education, groups had statistically significant differences (P=0.026) (Table 3).

**Table 3:** Paired comparison of mean changes in quality of life and general health scores in three groups of control, face to face and video education after the self-care program implementation

	**Group**	**Group**	**Differance of Mean±SD**	**Tukey’s post-hoc test result P value**
Quality of life	Control	Video education	-9.92±2.95	0.026
		Face to face education	-10.91±2.95	0.409
	Video education	Control	9.92±2.95	0.026
		Face to face education	-9.99±2.95	0.369
	Face to face education	Controln	10.91±2.95	0.409
		Video education	9.99±2.95	0.369
General health	Control	Video education	-9.62±3.65	0.026
		Face to face education	-4.67±3.65	0.409
	Video education	Control	9.62±3.65	0.026
		Face to face education	4.94±3.65	0.369
	Face to face education	Controln	4.67±3.65	0.409
		Video education	-4.94±3.65	0.369

## Discussion

The results of this study showed that among the three groups the demographic profile, such as gender, marital status, occupation, educational level, income, the number of dialysis per week, cause of disease, age and disease duration, was not significantly different. So, statistically significant differences in the dependent variable between control, face to face and video educational groups can be attributed to the effectiveness of interventions in those groups. The results of the present study showed that the general quality of life mean scores before the intervention in the three groups of control, video, and face to face education was not significantly differet; in other words, all the three groups were homogeneous in quality of life.

The quality of life mean scores after carrying out a self-care program in the video and face to face educational groups increased compared to the control group; this rise was statistically significant. Parde Zanjani and colleagues in a study on the impact of education on quality of life and physical problems in patients treated with maintenance hemodialysis reported similar results; thus, the self-care educational program for patients treated with hemodialysis is effective in reducing problems and promoting the general quality of life score in these patients.^32^ Narimani et al performed a study that concluded that providing adequate education in hemodialysis units by raising awareness can help improve energy levels, general health, physical function, mental health, andgeneral understanding about health, thereby raising the general quality of life in these patients; this is consistent with the results of the present study.^33^ The results of Choi et al in Seoul showed that patients under the caring plan through face to face education, compared with the control group, had statistically significant differences in knowledge and learning self-care practices.^34^

In the quasi-experimental study of Narooei and colleagues on the impact of the Orem Self Care Model on the quality of life in hemodialysis patients, it was indicated that after using this Model the quality of life in the samples in all aspects significantly improved; also the mean quality of life before and after the intervention increased significantly; this confirms the results of the present research.^35^ Tsay et al. in their study showed that reduction of stressors and depression was related to a higher quality of life.^36^

In this study, there was no difference in the quality of life of patients under the hemodialysis after education in two methods of face-to-face and video. Results of the study conducted by Hassanzadeh et al. showed the mean increase in attitudes on adherence to the diet in liquids in patients under dialysis had no statistically significant differences in two face to face and video educational groups; this is also consistent with the findings of the present study.^28^ Video education can also be as effective as face to face education on self-care of patients under hemodialysis, but confirmation on video education is different in various subjects.

The result of this study showed that after the intervention the mean scores for general health dimension among the three groups were different. The result of the study by Johansen et al. showed that the patients under dialysis who had received education about resistance exercises gained greater physical health.^37^ Chen and colleagues showed that 48 sessions of strength exercises twice a week improve muscle mass and muscle performance, thereby improving general health in hemodialysis patients.^38^ In a study performed by Campbell et al. aiming to evaluate nutritional interventions on quality of life in patients before dialysis, the researchers came to the conclusion that all dimensions of the quality of life with good nutrition as compared with malnourished patients were significantly different.^39^ Nurses can improve the quality of life in hemodialysis patients by using face to face and video educational methods. During the study, raising awareness of the research samples who used other sources than an than the planned educational program might have affected the research results; this was one of the limitations of the present study. The crises and possible problems that occurred to the patient or the family during the study, personal differences and psychological status of the patients have been other limitations of the study. The authors have no conflicts of interest.

In this study, each participant in face to face education group was trained only twice and the present study was conducted over a period of two months to compare the effect of the two educational programs on the dimensions of QOL in hemodialysis patients. Thus, one of its limitations was the short-term individual training and follow-up of patients. Further studies are required to investigate whether these early beneficial effects persist over longer durations or not. Another limitation of the present study was the relatively small number of patients; Therefore, further studies are recommended with larger sample sizes and longer follow-up period. The final limitation was that the present study only addressed hemodialysis patients; thus, its findings may not be generalizable to other groups of patients. 

## Conclusion

According to the findings of this research, it can be concluded that by carrying out both methods of face to face and video education, quality of life in hemodialysis patients rises although this rise has been slightly higher in face to face education. So, although verbal education is more effective and has advantages such as the educator’s presence and his interaction with patients, the results of this study showed that despite lack of active and present educator, video education has also been much effective in raising the patients’ quality of life. Because of the rising hemodialysis patients, being time consuming and having practical difficulties in face to face education, it is suggested that organizations should pay more attention to video education and invest more in this regard by using competent specialists. Nurses should educate the patients about self-care behaviors and remind them of the dangerous complications of abandoning these behaviors. 
